# 2-Hydroxymelatonin Induces Husk-Imposed Vivipary in the Transgenic Rice Overexpressing Melatonin 2-Hydroxylase

**DOI:** 10.3390/biom15101412

**Published:** 2025-10-04

**Authors:** Kyungjin Lee, Kyoungwhan Back

**Affiliations:** Department of Molecular Biotechnology, College of Agriculture and Life Sciences, Chonnam National University, Gwangju 61186, Republic of Korea; nicekj7@hanmail.net

**Keywords:** gibberellin, 2-hydroxymelatonin, *melatonin 2-hydroxylase*, seedling growth, transgenic rice (*Oryza sativa* L.), vivipary

## Abstract

Pre-harvest sprouting (PHS) reduces the quality and quantity of crop seeds. PHS can be imposed through the embryo or husk pathway of cereal crops. Most reported PHS seeds are imposed via the embryo pathway. Here, we generated transgenic rice plants overexpressing rice melatonin 2-hydroxylase (*OsM2H*), which catalyzes the hydroxylation of melatonin to 2-hydroxymelatonin (2-OHM). *OsM2H* overexpression (M2H-OE) showed PHS under paddy conditions. Germination assays revealed that intact seeds harvested at 26 and 36 days after heading (DAH) showed PHS, whereas dehusked seeds did not, indicating husk-imposed PHS. Overproduction of 2-OHM was observed in M2H-OE seeds compared to wild-type control. In addition, M2H-OE lines produced more hydrogen peroxide than the wild-type. 2-OHM-induced reactive oxygen species resulted in the induction of *OsGA3ox2*, a gibberellin (GA) biosynthesis gene, and repression of *OsGA2ox3*, a GA degradation gene, in caryopses at 2 DAH, but in the induction of the ABA degradation gene *OsABA8ox3* in intact seeds at 26 DAH. In addition, M2H-OE seedlings were longer and showed increased levels of hydrogen peroxide and *OsGA3ox2* expression versus the wild-type. This is the first report showing that 2-OHM can induce PHS via the husk pathway in rice seeds through the induction of GA biosynthetic and ABA degradation genes.

## 1. Introduction

Pre-harvest sprouting (PHS), also known as vivipary, occurs in the field in response to frequent rain and high temperatures during the seed maturation period, and seeds with PHS can no longer be used as food sources and sowing materials, resulting in serious yield losses, reduced seed viability, and grain quality degradation [1]. With global warming, many crops, including rice, are facing increased rates of PHS, which is estimated to result crop damage costing 1 billion dollars per year [2]. In particular, frequent rainfall and high temperatures during the maturation period promote PHS, and areas of PHS occurrence in rice are spreading throughout Southeast Asia [3]. PHS is caused by an imbalance between seed dormancy and seed germination, which is markedly influenced by the plant hormones abscisic acid (ABA) and gibberellin (GA) [4]. Seed dormancy is sustained by the presence of germination inhibitory compounds, including ABA, coumarin, dihydroflavonoids, and many other natural compounds [5]. In contrast, seed dormancy is released by seed germination-stimulating compounds, including GA, strigolactones, brassinosteroids (BRs), and hydrogen peroxide [6,7]. While many natural compounds play roles in seed dormancy and seed germination, changes in the GA/ABA ratio are key factors in determining germination or dormancy [8]. A higher GA/ABA ratio initiates germination, while a higher ABA/GA ratio maintains seed dormancy [9].

For example, PHS is greatly inhibited not only by an increased ABA level but also by enhancement of ABA signaling. Mutations in a number of ABA biosynthesis-related genes in rice (*Oryza sativa*), including those encoding the ABA biosynthesis cofactors *phytoene desaturase* [10], *9-cis-epoxycarotenoid dioxygenase3* (*NCED3*) [11], and *xanthine dehydrogenase* (*CNX6*) [12], hamper ABA biosynthesis, resulting in PHS. GA is known to promote PHS, and mutations of GA biosynthesis genes such as rice *GA20ox2* lead to PHS resistance [13], while overexpression of GA degradation genes, such as *GA2ox3*, results in inhibition of PHS [14]. Mutations in members of the *miR156* gene family have been shown to suppress PHS by downregulating multiple GA biosynthesis genes [15]. In addition to these major hormones, hydrogen peroxide (H_2_O_2_), acting as a signaling molecule, has been shown to play a pivotal role in seed germination in many plant species [7]. Exogenous treatment and increases in endogenous levels of hydrogen peroxide have been shown to induce GA rather than ABA, and thus to promote germination [16,17,18].

PHS is imposed by the rice embryo and/or seed coat (husk) [17]. The *Seed Dormancy1-2* mutant, with a mutation in *GA20ox2*, confers PHS resistance via an embryo-imposed pathway [13], whereas *Seed Dormancy7-1*, with a mutation in a gene encoding a *basic helix–loop–helix family transcription factor*, is associated with a combination of embryo- and husk-imposed PHS [19]. In addition, the ABA biosynthetic gene *NCED3* is involved in the embryo-controlled PHS pathway in rice [11]. To date, however, there have been no reports of PHS induced by rice husk alone.

2-Hydroxymelatonin (2-OHM), a melatonin metabolite catalyzed by melatonin 2-hydroxylase (M2H), was first discovered from rice [20]. It has been reported that four independent *M2H* genes exist in the rice genome [20]. Among four *OsM2H* genes, *OsM2H* gene (GenBank Accession No. AK119413) showing the highest M2H enzyme activity was selected as the major *OsM2H* gene in the rice genome. Initially, 2-OHM was thought to be a simple degradation product of melatonin as it is chemically much more inert than melatonin and its other hydroxylated derivatives, such as 6-hydroxymelatonin [21]. Contrary to speculation, however, 2-OHM is an emerging bioactive molecule that induces the production of reactive oxygen species (ROS) through respiratory burst nicotinamide adenine dinucleotide phosphate (NADPH) oxidase, resulting in premature senescence in Arabidopsis [22]. When melatonin or 2-OHM was administered to Arabidopsis leaves, melatonin induced the expression of antioxidant-related genes, such as *glutathione S-transferase 1* (*GST1*), and protein homeostasis-related genes including *heat shock protein 70* (*CpHSP70*) and *casein-degrading protease* (*ClP*), whereas 2-OHM induced the expression of cell death- and senescence-related genes such as *ABA-insensitive 5* (*ABA5*) and *ethylene-responsive transcription factor* (*ERF*), indicating that melatonin and 2-OHM have different biological roles. In addition, Lee and Back [23] recently reported that 2-OHM treatment promoted Arabidopsis seed germination, with increases in ROS production and GA biosynthetic gene expression. To confirm whether 2-OHM also has a germination-promoting effect in rice, we attempted to generate transgenic rice overexpressing *OsM2H* under the control of the maize ubiquitin promoter, but failed due to failure of somatic embryogenesis [24]. This was probably due to ROS production caused by *OsM2H* overexpression [22].

In this study, stable *OsM2H* overexpressing transgenic rice plants were developed and used to evaluate the functional role of 2-OHM in rice. In order to alleviate the toxic effects of *OsM2H* overexpression during the process of somatic embryo formation, the rice *OsM2H* gene was overexpressed under the control of the *CaMV 35S* promoter, which has lower promoter activity than the maize ubiquitin promoter in the context of rice [25]. As expected, transgenic rice plants overexpressing rice *OsM2H* were successfully generated. *OsM2H*-overexpressing (M2H-OE) seeds exhibited PHS, which was ascribed to husk-imposed germination as dehusked seeds had an identical germination rate to non-transformed wild-type (WT) controls. Enhanced levels of 2-OHM in the M2H-OE seeds were coupled with increased superoxide levels. Pericarps from 2 days after heading (DAH) showed that the GA biosynthetic gene *OsGA3ox2* was upregulated, whereas the GA degradation gene *OsGA2ox3* was downregulated, in M2H-OE compared to wild-type. These observations implicated 2-OHM in rice seed PHS.

## 2. Materials and Methods

### 2.1. Generation of Transgenic Rice Plants Overexpressing Rice OsM2H Gene

As for the ectopic overexpression of rice *OsM2H* (GenBank accession number AK119413) under the control of *CaMV 35S* promoter in rice genome, we utilized a gateway binary vector pH2GW7 [26]. In brief, the pDONR221-*OsM2H* entry vector which was previously constructed [24,27] was recombined with the pH2GW7 destination vector via LR recombination to yield pH2GW7-OsM2H, which was then transformed into *Agrobacterium tumefaciens* strain LBA4404. Japonica rice cultivar called Dongjin was employed to obtain embryogenic calli for Agrobacterium-mediated rice transformation. After coculture procedure between calli and Agrobacterium, transgenic seedlings grown in the presence of hygromycin antibiotic were generated and selected for T_0_, T_1_, and T_2_ seed production [24].

### 2.2. Plant Materials and Growth Conditions in the Field

T_2_ homozygous transgenic rice seeds were sown and grown in a paddy field of Chonnam National University (53 m a.s.l.; 35°09′ N and 126°54′ W), Gwangju, Republic of Korea. Fertilizer was applied at 70 N/40 P/70 K kg/ha. Seeds were collected at different days after heading (DAH) in the paddy field for investigating PHS.

### 2.3. Germination Analysis

For germination assays, healthy, uniform seeds (both intact and dehusked) freshly collected 26 and 36 days after heading (DAH) were surface-sterilized with 100% ethanol for 30 s and then with 2% sodium hypochlorite for 5 min. The resulting seeds were rinsed three times in deionized water for 5 min each. Twenty-five seeds were placed in 15 mL of distilled water in a Petri dish plate. The seeds were germinated at 28 °C under LD (14 h light/10 h dark) conditions. Germination percentage was calculated at different time intervals. A seed was considered germinated when the length of the newly emerged coleoptile exceeded 1 mm. Each treatment consisted of three replicates of 25 seeds.

### 2.4. 2-Hydroxymelatonin Measurement from the M2H-OE Seeds

Intact seeds and husks (0.1 g) collected at 26 DAH were used for the measurement of 2-hydroxymelatonin concentrations. Intact seeds were imbibed for 24. The imbibed intact seeds and husks were extracted with 1 mL chloroform, centrifuged at 12,000× *g* for 10 min, and the supernatant (200 µL) was evaporated and dissolved in 0.1 mL of 40% methanol. A 10 µL aliquot was injected into a high-performance liquid chromatography (HPLC) equipped with a UV detector (Waters, Milford, MA, USA) at 254 nm. 2-OHM was separated on a Sunfire C18 column (Waters; 4.6 × 150 mm) at a flow rate of 0.25 mL/min for 25 min using an isocratic elution method with 35% MeOH in 0.3% TFA as previously described [24].

### 2.5. Nitrotetrazolium Blue Staining

Superoxide (O_2_^−^) was detected via in situ histochemical staining using nitrotetrazolium blue (NBT). Seeds collected after 26 DAH were imbibed in water for 48 h and immersed in a solution containing 0.1% NBT (10 mM MES, pH 6.8) for 1 day, and then stored in ethanol (96%). Superoxide anions were visualized as precipitates of dark blue insoluble formazan compounds.

### 2.6. Total RNA Isolation and Reverse Transcription–Polymerase Chain Reaction (RT-PCR)

Total RNA was isolated from caryopses and intact seeds collected at 2 and 26 DAH in the field, respectively, using a Ribospin Plant Kit (GeneAll Biotechnology Co., Seoul, Republic of Korea). First-strand cDNA was synthesized from 1 µg total RNA using RevertAid Reverse Transcriptase (Thermo Scientific Fermentas, St. Leon-Rot, Germany) and oligo(dT) primers (Promega, Madison, WI, USA). PCR amplification was then performed using 0.2 µL of the reverse transcription reaction as a template, and the expression of germination-related genes was analyzed by RT-PCR. The rice *ubiquitin-5 gene* (*OsUBQ5*) was used as a loading control. The primer sequences are as follows: *OsM2H* (forward 5′-CCG AGT TCT TCC AGC TAT CG-3′, reverse 5′-GGC GTA CTC ACC CAT TTT CT-3′), *OsGA3ox2* (forward 5′-GCT ACA CCT TCT CCC CTT CC-3′, reverse 5′-CTC GGG TAC CAG TTG AGG TG-3′), *OsGA2ox3* (forward 5′-TGG TGG CCA ACA GCC TAA AG-3′, reverse 5′-TGG TGC AAT CCT CTG TGC TAA C-3′), *OsNCED2* (forward 5-GGT ATG GAA ACG AGG ATA GTG-3′, reverse 5′-TGC TTA TTG TTG TGC GAG AAG-3′), *OsABA8ox3* (forward 5′-AGT ACA GCC CAT TCC CTG TG-3′, reverse 5′-ACG CCT AAT CAA ACC ATT GC-3′), *OsABI5* (forward 5′-CAA GGC GGT CCT ATG ATG TT-3′, reverse 5′-ATC CAG GAC TCA CGA CAA CC-3′), and *OsUBQ5* (forward 5′-CCG ACT ACA ACA TCC AGA AGG AG-3′, reverse 5′-AAC AGG AGC CTA CGC CTA AGC-3′). The resulting PCR products were electrophoresed on ethidium bromide gels and photographed under ultraviolet (UV) light. As for the real time PCR analysis (qPCR), a Mic qPCR Cycler system (Bio Molecular Systems, Queensland, VIC, Australia) with the Luna Universal qPCR Master Mix (New England Biolabs, Ipswich, MA, USA) was utilized as described previously [24].

### 2.7. Seedling Growth and Hydrogen Peroxide Measurement

M2H-OE (T_2_) and wild type rice seeds were dehusked, surface-sterilized and germinated on half-strength Murashige and Skoog (MS) medium (MB Cell, Seoul, Republic of Korea) in vertically oriented polystyrene square dishes (SPL Life Science, Pocheon-si, Republic of Korea). The growing conditions for rice seedlings were 28 °C under a 12/12-h light/dark cycle, at a photosynthetic photon flux density of 100 µmol m^−2^ s^−1^ for 7 days. These seedlings were harvested to measure their length and for further molecular analyses. Hydrogen peroxide contents were quantified by OxiTec™ Hydrogen Peroxide/Peroxidase (H_2_O_2_) Assay Kit (Biomax, Guri-si, Republic of Korea).

### 2.8. Statistical Analysis

Data were analyzed by analysis of variance using IBM SPSS Statistics 23 software (IBM Corp. Armonk, NY, USA). Means with different letters indicate significantly different values at *p* < 0.05 according to Tukey’s post hoc honestly significant difference (HSD) test. Data are presented as mean ± standard deviation.

## 3. Results

### 3.1. Transgenic Rice Plants Overexpressing Rice M2H Under the Control of the CaMV 35S Promoter

**Figure 1:**
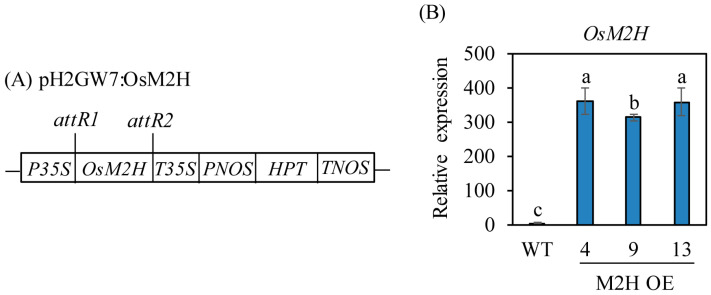
Generation of *Oryza sativa M2H* (*OsM2H*) overexpression transgenic rice plants. (**A**) Schematic diagram of pH2GW7:OsM2H binary vector. (**B**) Quantitative real time PCR (qPCR) analyses of transgenic and wild type (WT) seeds at 26 days after heading (DAH) in a paddy field. WT, non-transformed wild type control; *OsUBQ5*, *rice ubiquitin 5* gene. GenBank accession number are *OsUBQ5* (AK061988) and *melatonin 2-hydroxylase* (*OsM2H*; AK119413). Different letters indicate significant differences among lines (Tukey’s HSD; *p* < 0.05). *P35S, cauliflower mosaic virus (CaMV) 35S promoter; T35S, CaMV 35S terminator; PNOS, nopaline synthase promoter; HPT, hygromycin phosphotransferase; TNOS, nopaline synthase terminator.*

Ectopic overexpression of rice *M2H* (*OsM2H*) under the control of the high-activity maize ubiquitin promoter led to embryogenic lethality during somatic embryogenesis, resulting in failure to generate rice M2H-OE transgenic rice plants [24]. To modulate its expression level, we attempted to express *OsM2H* under the control of the *CaMV 35S* promoter to avoid the toxic effect of *OsM2H* overexpression (Figure 1A). We successfully generated stable M2H-OE transgenic rice plants without embryogenesis lethality. First, a total of 13 independent T_0_ transgenic lines were generated through Agrobacterium-mediated transformation. From among these lines, seeds from three homozygous T_2_ lines were further selected to examine the gain-of-function effects of *OsM2H* in the rice genome. To verify the ectopic overexpression of *OsM2H* mRNA, quantitative real-time PCR (qPCR) analysis was performed in M2H-OE transgenic rice plants. Rice *OsM2H* mRNA was detected in total RNA isolated from imbibed intact rice seeds for 24 h harvested 26 DAH from the paddy field. As shown in Figure 1B, rice *OsM2H* mRNA was significantly overexpressed in M2H-OE transgenic lines, with an average of 67-fold higher expression compared to the wild-type (WT) control. These data clearly suggested that the *CaMV 35S* promoter efficiently overexpressed rice *OsM2H* in the rice genome.

### 3.2. Enhanced Pre-Harvest Sprouting of M2H-OE Panicles

**Figure 2:**
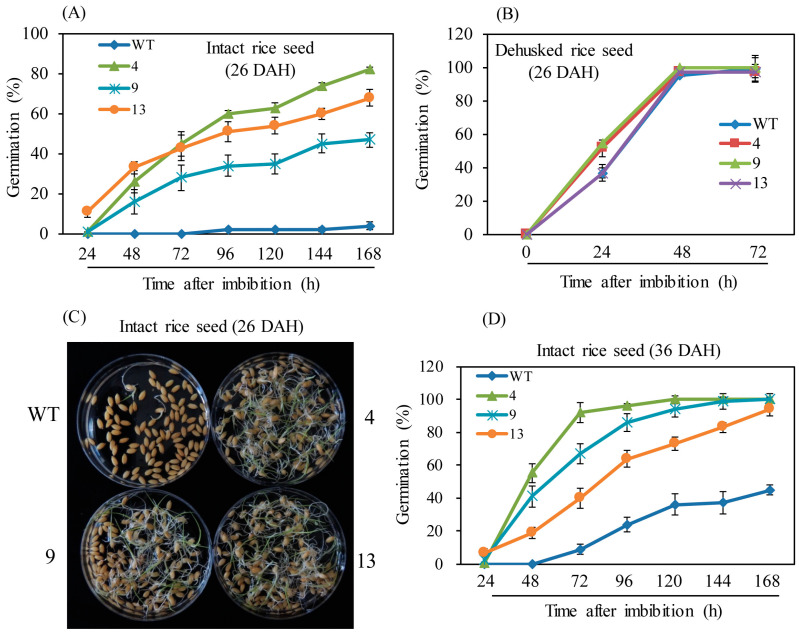
(**A**) Germination rate of intact rice seeds at 26 DAH. (**B**) Germination rate of dehusked rice seeds at 26 DAH. (**C**) Photograph of germinated intact rice seeds at 26 DAH. (**D**) Germination rate of intact rice seeds at 36 DAH. Seeds were freshly harvested from a paddy field. WT, non-transformed wild type control.

Spikelets were harvested under field conditions at 26 and 36 DAH, and germination rates were monitored over time. First, germination rates were tested in intact rice seeds at 26 DAH. While the wild-type showed less than 2% germination at 168 h imbibition, the M2H-OE lines showed an average germination rate of 60%, indicating significantly suppression of rice seed dormancy by *OsM2H* overexpression (Figure 2A,C). Unlike intact rice seeds, the dehusked seeds of the M2H-OE lines showed germination rates similar to the wild-type (Figure 2B). There was no difference in rice seed dormancy between the M2H-OE lines and the wild-type when the seeds were dehusked. These observations suggested that the PHS symptoms occurring in the M2H-OE lines were caused by the husk, not the embryo or endosperm. Unlike the 26-DAH spikelets, the wild-type spikelets harvested at 36 DAH had a higher germination rate of approximately 40% at 168 h after imbibition, while the corresponding M2H-OE spikelets showed an average germination rate of 90% (Figure 2D).

**Figure 3:**
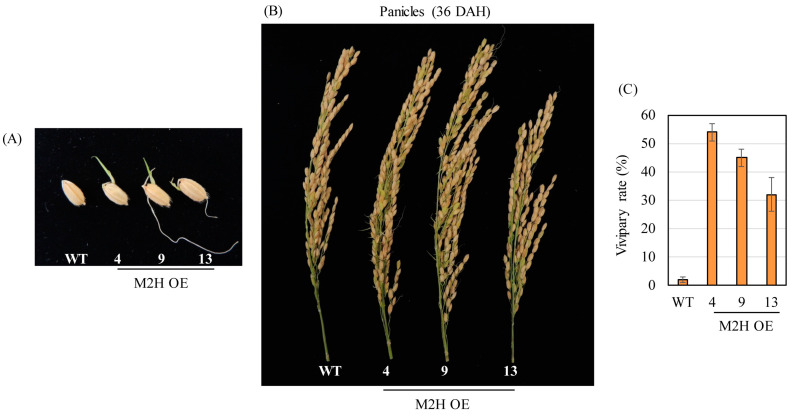
(**A**) Representative caryopses showing a pre-harvest sprouting (PHS) at 36 DAH. (**B**) Photograph of panicles at 36 DAH. (**C**) Measurement of vivipary rate at 36 DAH from a paddy field. WT, non-transformed wild type control.

When the T_2_ homozygous M2H-OE lines were grown under paddy conditions, the spikelets of the M2H-OE lines exhibited PHS at 36 DAH (Figure 3A,B). Under field conditions, the M2H-OE line exhibited an average of 44% PHS, whereas the wild type showed only about 2% PHS (Figure 3C). These observations indicated that *OsM2H* overexpression induced PHS in rice. Taken together, these results indicated that *OsM2H* overexpression in rice conferred vivipary, imposed by the husk rather than the embryo or endosperm.

### 3.3. Measurements of 2-Hydroxymelatonin and Superoxide in Intact Seeds

**Figure 4:**
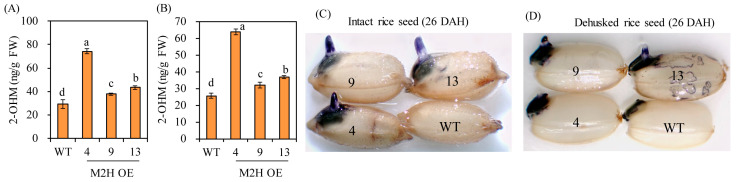
(**A**) 2-Hydroxymelatonin (2-OHM) levels from imbibed intact seeds for 24 h. (**B**) 2-OHM levels in husks. (**C**) Superoxide staining of intact rice seeds. (**D**) Superoxide staining of dehusked rice seeds. Seeds harvested at 26 DAH were used in this experiment. Nitrotetrazolium blue (NBT) staining was conducted to assess O_2_^−^ accumulation. Values are means ± SD (n = 3). Different letters indicate significant differences among lines (Tukey’s HSD; *p* < 0.05). WT, non-transformed wild type control.

To determine whether *OsM2H* overexpression is associated with 2-OHM production, we collected intact seeds and hulls at 26 DAH. After imbibing intact seeds for 24 h, 2-OHM levels were quantified by high-performance liquid chromatography (HPLC). As shown in Figure 4, 2-OHM levels were higher in both intact seeds and husks of the M2H-OE line than in the wild-type control (Figure 4A,B). As 2-OHM was reported to induce superoxide production in Arabidopsis [23], we investigated superoxide levels by staining with nitro blue tetrazolium (NBT). Dark-blue precipitates were detected in the 24-h imbibed intact seeds of M2H-OE lines, but no staining was seen in wild-type intact seeds (Figure 4C). In contrast, superoxide was observed in all dehusked seeds, but levels were much higher in M2H-OE than in wild-type seeds (Figure 4D). These results suggested that 2-OHM is also coupled with ROS production in rice, as reported in Arabidopsis [23].

### 3.4. Transcript Expression Profiles of Germination-Related Genes in Intact Seeds

**Figure 5:**
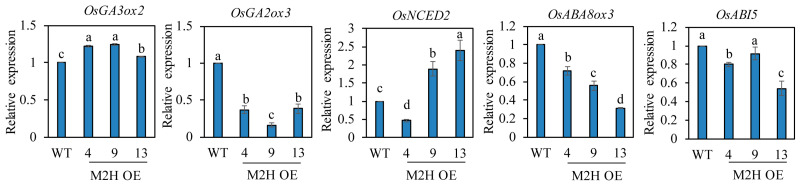
qPCR analysis of GA and ABA-related genes from caryopses harvested at 2 DAH. GenBank accession number are *OsUBQ5* (AK061988), *OsGA3ox2* (AB056519), *OsGA2ox3* (AB092485), *OsNCED2* (AY828898), *OsABA8ox3* (AK288402), and *OsABI5* (EF199631). Values are means ± SD (n = 3). Different letters indicate significant differences among lines (Tukey’s HSD; *p* < 0.05). WT, non-transformed wild type control.

**Figure 6:**
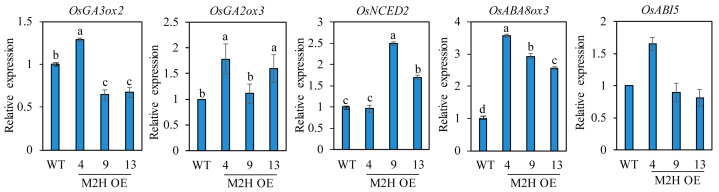
qPCR analysis of GA and ABA-related genes from imbibed intact rice seeds harvested at 26 DAH. GenBank accession number are described in Figure 5 legend. Values are means ± SD (n = 3). Different letters indicate significant differences among lines (Tukey’s HSD; *p* < 0.05). WT, non-transformed wild type control.

Seed dormancy and germination are closely associated with the relative levels of ABA and GA, with a high ABA/GA ratio increasing seed dormancy and a high GA/ABA ratio inducing seed germination. It is well known that the GA biosynthesis gene *OsGA3ox2,* and the GA degradation *OsGA2ox3* play a pivotal role in regulating GA levels in rice seed germination [28]. The ABA biosynthesis gene *OsNCED2* and the ABA catabolic gene *OsABA8ox3* are known to play important roles in PHS-related ABA biosynthesis and degradation during rice grain development [11]. To measure these hormone levels indirectly, we quantified their mRNA transcript levels. First, we measured these transcript levels in spikelets harvested at 2 DAH from the paddy field (Figure 5). *OsNCED2*, *OsABA8ox3,* and *OsABI5* levels were inconsistent even in the M2H-OE lines. However, *OsGA3ox2* expression was higher, and *OsGA2ox3* expression was lower, in the M2H-OE lines than wild-type controls, indirectly suggesting that GA levels increase in the M2H-OE line during the early stages of seed development. ROS has been reported to induce ABA catabolism and GA biosynthesis during seed germination [29]. Because of the role of 2-OHM-induced ROS generation, we expected to see induction of GA biosynthetic genes, along with ABA degradation genes in the spikelets of M2H-OE at 2 DAH. However, only the GA-related genes were observed. This result led us to assume that ABA-related genes may be involved in a later stage of seed development, such as 26 DAH. Therefore, we next measured their transcript levels in imbibed intact rice seeds at 26 DAH. The transcript levels of both *OsGA3ox2* and *OsGA2ox3* genes showed marked variability among the M2H-OE lines, indicating inconsistency in the increase in GA level among lines (Figure 6). With regard to ABA, the transcript levels of the ABA biosynthesis gene *OsNCED2* were elevated in some M2H-OE lines, such as lines 9 and 13, but were similar between line 4 and the wild-type. In contrast, the ABA degradation gene *OsABA8ox3* showed higher transcript levels in the M2H-OE lines than in wild-type controls. The levels of OsABI5 were variable among the M2H-OE lines. Thus, it was concluded that the major cause of vivipary in the M2H-OE lines at 26 DAH when judged by gene expression profiles is attributable to the decrease of ABA in seeds.

### 3.5. M2H-OE Lines Exhibited Enhanced Seedling Growth

**Figure 7:**
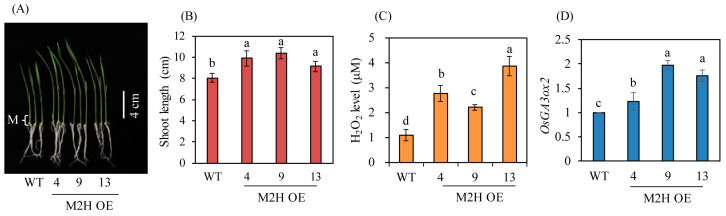
Enhanced seedling growth in M2H-OE transgenic rice plants. (**A**) Photograph of 7-day-old rice seedlings. (**B**) Shoot length. (**C**) Hydrogen peroxide (H_2_O_2_) contents. (**D**) qPCR analysis of *OsGA3ox2*. Meristemic region of 7-day-old seedlings were used for measuring Hydrogen peroxide and *OsGA3ox2* levels. Different letters indicate significant differences among the lines (Tukey’s HSD; *p* < 0.05). M indicates meristemic region. WT, non-transformed wild type control.

Increased endogenous GA production is closely coupled with enhanced seedling growth in rice [15]. To verify increases in GA levels in the M2H-OE lines, surface-sterilized dehusked seeds were sown in MS medium and cultured for 7 days. The M2H-OE lines showed enhanced seedling growth relative to wild-type, with an average increase of about 20% in seedling length (Figure 7A,B). Meristematic tissues, which are the main sites of action of GA, were collected, and hydrogen peroxide and *OsGA3ox2* transcript levels were measured. Hydrogen peroxide levels were higher in the M2H-OE lines than in wild-type controls (Figure 7C). These data were consistent with those in Arabidopsis, in which 2-OHM was shown to induce hydrogen peroxide production [23]. In addition, the key GA biosynthetic gene *OsGA3ox2* was expressed at higher levels in the M2H-OE lines than in wild-type controls (Figure 7D). Taken together, these observations suggest that GA synthesis was increased in both the husks and seedlings of the M2H-OE line compared to the wild type, even though GA contents were not directly measured. Therefore, the occurrence of PHS in the M2H-OE line appears to be due to increased 2-OHM synthesis, followed by increased ROS production and upregulation of the GA biosynthetic gene *OsGA3ox2* and the ABA degrading gene *ABA8ox3*.

## 4. Discussion

PHS is a phenomenon in which rice spikelets germinate while the panicle is still attached to the plant stem, also known as vivipary [2]. PHS is usually caused by the release of seed dormancy triggered by either decreases in the levels of seed dormancy-inducing compounds or increases in the levels of seed dormancy-inhibiting compounds [5,6]. Although many chemicals have been implicated in seed dormancy, their ultimate mode of action converges on GA and ABA, which are antagonistically associated with seed dormancy. On the other hand, chemicals or proteins that affect GA or ABA sensitivity or signaling also greatly affect seed dormancy [30,31,32]. For example, *MAPKKK63* overexpression induced a viviparous phenotype in both rice and Arabidopsis by reducing ABA sensitivity [32]. Two single recessive viviparous mutants of rice, *riv1* and *riv2*, have lower sensitivity to ABA [30]. Endosperm sugar accumulation also leads to PHS in rice by suppression of the expression of ABA signaling genes, such as *ABI3* and *ABI5* [31]. Many quantitative trait loci (QTLs) associated with PHS have been discovered through molecular genetic analyses [2], and the corresponding genes have been identified [2,31]. In addition, several novel genes that cause PHS have been identified by transcriptomic analysis [33,34,35]. Most of these PHS-induced genes are associated either directly or indirectly with the GA and ABA pathways [3]. Therefore, many target genes involved in the ABA and GA pathways are being engineered through genome editing and RNA interference (RNAi) technologies, with the aim of generating PHS-resistant rice [10,36].

Melatonin, first identified as a pineal neurohormone in animals, was also discovered in plants in 1995 [37]. In contrast to its role as a hormone in animals, melatonin acts as a biotic stimulant in plants that coordinates various physiological functions, including primary growth, secondary growth, and defense against biotic and abiotic stresses [38]. Although a candidate melatonin receptor has been reported to have been cloned from plants [39], its authenticity remains controversial [23]. Melatonin has been considered to have various biological activities in plants [21,40]. However, recent reports have shown that melatonin is enzymatically converted into other biological molecules, such as 2-OHM and cyclic 3-hydroxymelatonin (3-OHM) [40]. *OsM2H* was first cloned from rice, and its product, 2-OHM, was found to be present in plants in much higher amounts than melatonin [20,27]. In addition, 3-OHM levels were shown to be at least three orders of magnitude higher than those of melatonin in rice [40]. These data suggest that melatonin is rapidly converted to 2-OHM and 3-OHM, which are the major compounds of melatonin in plants [40]. As a result, exogenous melatonin treatment of plants produces a mixture of melatonin, 2-OHM, and 3-OHM, making it difficult to assess the function of melatonin. For example, although melatonin treatment has been reported to induce seed germination in plants [41,42], this is known to be due to the effect of 2-OHM and not melatonin itself [23]. In addition, melatonin treatment has been reported to induce ROS in Arabidopsis [39], but this effect is also due to 2-OHM and not melatonin [22]. Recently, it was reported that an Arabidopsis double-knockout mutant lacking *M2H* and *M3H* (*m2hm3h*) exhibited dwarfism, characterized by a short hypocotyl length and reduced biomass compared to wild-type control [40]. The stunted growth of *m2hm3h* was attributed to suppression of *PHYTOCHROME INTERACTING FACTOR* (*PIF*)*4* and *PIF5*, which are pivotal transcription factors for skotomorphogenic seedling growth. In accordance with the expression of *PIF4* and *PIF5*, *M2H* and *M3H* expression peaked at night, but their expression was significantly suppressed in *PIF4PIF5* double mutant. *PIF4*- and *PIF5*-mediated *M2H* and *M3H* expression led to enhanced expression of GA and BR biosynthesis genes, followed by the promotion of skotomorphogenic seedling growth, indicating that 2-OHM and 3-OHM together act as dark signaling molecules for plant growth.

The mechanism by which 2-OHM induces PHS in rice seeds is believed to be similar to the role of 2-OHM in inducing Arabidopsis leaf senescence and germination through ROS generation [22,23]. ROS generation by 2-OHM is known to be dependent on respiratory burst NADPH oxidase in Arabidopsis [22]. Similar to the 2-OHM-mediated enhancement of germination in Arabidopsis, M2H-OE transgenic rice seeds produced more 2-OHM than wild-type control (Figure 4A,B), followed by increased ROS production (Figure 4C,D). In general, ROS such as hydrogen peroxide can break seed dormancy and promote germination in many plant species [17]. In Arabidopsis, exogenous hydrogen peroxide increased ABA degradation and enhanced expression of genes involved in GA synthesis [29]. In barley, genes related to GA biosynthesis were upregulated, while ABA-related genes were not. These observations indicated a species-specific ABA/GA gene expression profile induced by hydrogen peroxide [43]. Seed germination induced by ROS was completely inhibited by treatment with diphenyleneiodonium (DPI), an NADPH oxidase inhibitor, suggesting that ROS synthesis is mediated by NADPH oxidase in the cell membrane [16,17]. Similarly, 2-OHM-mediated seed germination enhancement was abolished by DPI treatment in Arabidopsis [23].

PHS is imposed by one or more of the embryo, endosperm, and husk [19]. Most reports on PHS to date were related to the embryo and/or endosperm [11,13,44]. On the other hand, 2-OHM-mediated PHS is imposed by the husk, as there was no difference in germination between dehusked wild-type and dehusked M2H-OE seeds. In this respect, 2-OHM plays an interesting role in PHS in rice seeds (Figure 3). Taken together, these observations suggest that the increase in PHS induced by 2-OHM overexpression in M2H-OE lines is coupled with increased reactive oxygen species (ROS) production, which induces the expression of genes related to ABA degradation and GA biosynthesis. This differential expression of GA- and ABA-related genes in M2H-OE seeds may lead to an increase in the GA/ABA ratio, which induces PHS (Figure 5 and Figure 6). Direct measurement of GA and ABA contents in the husks of M2H-OE lines will clarify the actual mechanism by which 2-OHM induces PHS in rice seeds.

In summary, this study revealed a novel physiological role of 2-OHM as an inducer of PHS in rice plants. Although 2-OHM was initially considered a simple degradation product of melatonin in plants [21], it has now been shown to exhibit a wide array of biological functions. The main function of 2-OHM is the induction of ROS, possibly via NADPH oxidase, which in turn accelerates leaf senescence and germination in Arabidopsis [23]. Similarly, 2-OHM together with 3-OHM promotes night seedling growth by inducing GA and BR biosynthesis [40]. Similar to the enhanced germination effect, endogenous 2-OHM overproduction in M2H-OE transgenic rice plants caused PHS through the husk-imposed pathway. Based on these results, 2-OHM is considered to be a novel plant natural compound in the husk of rice seeds that induces PHS. Therefore, *OsM2H* has the potential to be a valuable genetic resource for PHS resistance and stable seed production in the coming era of global warming.

## 5. Conclusions

Here, we successfully generated transgenic rice plants overexpressing rice *OsM2H* under the control of the *CaMV 35S* promoter. M2H-OE plants showed PHS under paddy conditions. Germination assays showed that intact seeds harvested at 26 and 36 DAH showed PHS, whereas dehusked seeds did not, indicating husk-imposed PHS. In addition, M2H-OE lines produced higher levels of hydrogen peroxide than WT controls. 2-OHM-induced ROS resulted in the induction of *OsGA3ox2*, a gibberellin (GA) biosynthesis gene, and repression of *OsGA2ox3*, a GA degradation gene, in caryopses at 2 DAH as well as the induction of *OsABA8ox3*, a ABA degradation gene, in the intact seeds harvested at 26 DAH. This is the first report that 2-OHM can induce PHS via the husk pathway in plants. Although we did not measure the contents of GA and ABA, it is clear that *OsM2H* is involved in PHS, and its knockout mutant is expected to be valuable genetic resources for PHS resistance.

## Data Availability

The original contributions presented in this study are included in the article. Further inquiries can be directed to the corresponding author.
